# Assessment of RAS-RAF-MAPK Pathway Mutation Status in Healthy Skin, Benign Nevi, and Cutaneous Melanomas: Pilot Study Using Droplet Digital PCR

**DOI:** 10.3390/ijms25042308

**Published:** 2024-02-15

**Authors:** Elena-Georgiana Dobre, Luciana Nichita, Cristiana Popp, Sabina Zurac, Monica Neagu

**Affiliations:** 1 Doctoral School, Faculty of Biology, University of Bucharest, 050095 Bucharest, Romania; dobregeorgiana_95@yahoo.com; 2 “Victor Babes” National Institute of Pathology, 050096 Bucharest, Romania; luciana.nichita@yahoo.com (L.N.); brigaela@yahoo.com (C.P.); sabina_zurac@yahoo.com (S.Z.); 3 Colentina Clinical Hospital, 020125 Bucharest, Romania; 4 Department of Pathology, Faculty of Dental Medicine, “Carol Davila” University of Medicine and Pharmacy, 020021 Bucharest, Romania

**Keywords:** BRAF V600E mutation, NRAS G12/G13 mutation, melanoma, nevi

## Abstract

In the present study, we employed the ddPCR and IHC techniques to assess the prevalence and roles of *RAS* and *RAF* mutations in a small batch of melanoma (*n* = 22), benign moles (*n* = 15), and normal skin samples (*n* = 15). Mutational screening revealed the coexistence of *BRAF* and *NRAS* mutations in melanomas and nevi and the occurrence of *NRAS* G12/G13 variants in healthy skin. All investigated nevi had driver mutations in the *BRAF* or *NRAS* genes and elevated p16 protein expression, indicating cell cycle arrest despite an increased mutational burden. *BRAF* V600 mutations were identified in 54% of melanomas, and *NRAS* G12/G13 mutations in 50%. The *BRAF* mutations were associated with the Breslow index (BI) (*p* = 0.029) and TIL infiltration (*p* = 0.027), whereas the *NRAS* mutations correlated with the BI (*p* = 0.01) and the mitotic index (*p* = 0.04). Here, we demonstrate that the “young” ddPCR technology is as effective as a CE-IVD marked real-time PCR method for detecting *BRAF* V600 hotspot mutations in tumor biopsies and recommend it for extended use in clinical settings. Moreover, ddPCR was able to detect low-frequency hotspot mutations, such as *NRAS* G12/G13, in our tissue specimens, which makes it a promising tool for investigating the mutational landscape of sun-damaged skin, benign nevi, and melanomas in more extensive clinical studies.

## 1. Introduction

Cutaneous melanoma (CM) is a malignant melanocytic tumor, representing less than 5% of all skin cancers. Despite its low prevalence, it is the most severe skin neoplasia, accounting for 80% of skin cancer-associated deaths, especially among the young adult population [1]. In Europe and the USA, the incidence of CM has increased four-fold in the last 30 years [2]. Established CM risk factors include ultraviolet (UV) radiation exposure, phenotypic traits with a solid genetic component, such as fair skin complexion and red or blonde hair, and an increased number of common and atypical melanocytic nevi, alongside a positive family history for melanoma [3]. All these conditions subside to chronic inflammation events that favor tumorigenesis [4]. Death from CM is mainly due to distant metastasis or drug resistance [5]. The 5-year relative survival rate for advanced CM is 25% and falls to 10% surviving at ten years [6,7]. Notably, more than 95% of CM cases can be successfully treated if they are discovered in the early stages, suggesting that it is critical to understand this disease’s biology to improve its clinical management [7]. CM arises from the neoplastic transformation of neural crest stem cell-derived melanocytes [8]. Recent studies evaluating the mutational status of human melanomas have revealed an increased mutation load associated with the molecular signatures of UV damage [9,10,11]. In line with these observations, the latest World Health Organization (WHO) classification of skin tumors highlights the existence of two major melanoma subtypes—the low cumulative sun damage (low CSD) subtype and the high cumulative sun damage (high CSD) subtype, emphasizing solar exposure as the main pro-inflammatory carcinogen involved in CM formation [12]. Melanomas that arise against a background of low-CSD typically harbor *BRAF* V600E mutations (and rarely *NRAS* Q61 mutations) and originate from benign nevi. Most of these melanomas occur on the trunk and extremities of adults between the ages of 20 and 60 [13]. The conversion of *BRAF* V600E positive nevi to full-blown melanomas is achieved through a plethora of genetic and epigenetic alterations [14,15]. Additional mutations, such as in cyclin-dependent kinase inhibitor 2A (*CDKN2A*) or the promoter of the human telomerase reverse transcriptase (*hTERT*) and chromosomal copy number aberrations have been frequently reported in low-CSD melanoma [16]. By contrast, high-CSD melanomas do not harbor *BRAF* V600E mutations but present other mitogen-activated protein kinase (*MAPK*) pathway mutations, such as *BRAF* V600K, *NRAS* G12/G13, or *KIT* mutations, or inactivation of the negative regulators of *Ras*, such as *NF1* or *RASA2*. Melanoma in situ is considered the main precursor lesion of high-CSD tumors, as pre-existing nevi are not commonly observed in those tumors. Finally, high-CSD melanomas encompass the lentigo maligna and desmoplastic melanoma subtypes, whereas superficial spreading melanomas mainly represent low-CSD tumors [13]. The 2018 WHO classification of melanomas intends to incorporate all the causal mechanisms and clinical/histopathological parameters of cutaneous tumors to improve the diagnosis and surveillance in the clinical setting [17].

The genomic profiling of melanoma tumors revealed several recurrent mutations involved in their pathogenesis and evolution, such as *BRAF*, *NRAS*, and *KIT*, contributing to these tumors’ genomic subtyping [18]. At the molecular level, it is supposed that somatic mutations in *NRAS* and *BRAF*, which occur mutually exclusive, are critical in this multistep development of melanoma. These mutations cause the constitutive activation of the serine-threonine kinases in the *ERK–MAPK* pathway, which results in augmented tumor proliferation and growth [19]. The assessment of *BRAF* mutational status is critical, as it indicates those tumors amenable to Food and Drug Administration (FDA)-approved targeted therapies, such as BRAF inhibitors (BRAFi: vemurafenib, dabrafenib) and MEK inhibitors (MEKi: trametinib, cobimetinib) [20]. However, the exact role of *BRAF* mutations in the initiation or progression of melanoma is still controversial. The *BRAF* gene encodes a protein that plays a pivotal role in *MAPK* pathway activation, contributing to cellular growth, differentiation, survival, and proliferation [21]. *BRAF* mutations are usually identified in the codon 600 of the *BRAF* gene and are reported in 40–60% of melanoma cases [22,23,24,25]. The *BRAF* V600E mutation, which involves the substitution of glutamic acid with valine at codon 600 (*BRAF* V600E: nucleotides 1799 T > A; codon GTG > GAG), is the most frequent mutation reported in CM. This mutation is present in over 90% of BRAF-positive melanomas [26]. Less common modifications include substitutions of valine for lysine (V600K), arginine (V600R), leucine (V600M), or aspartic acid (V600D) [22]. Many clinical studies have highlighted that the *BRAF* V600E mutation is usually identified in younger patients and depicted in tumors on skin subjected to intermittent sun exposure, such as the upper limbs, trunk and, less frequently, the head and neck. Wild-type melanoma tumors are typically found on the lower limbs [27,28]. Nonetheless, melanomas with *BRAF* V600 mutations respond well to current FDA-approved BRAFi, as well as combined BRAF/MEK inhibitor therapy [29]. Moreover, the ratio of mutant/wild-type alleles of *BRAF* was reported to be a potential biomarker for prognosis in this hard-to-treat disease, as an increased ratio was associated with superior clinical outcomes and reasonable response to BRAFi therapy [30].

The *RAS* gene family includes *KRAS*, *NRAS*, and *HRAS* proto-oncogenes, which encode several small GTPases involved in cell growth and proliferation. Mutated *NRAS* is the second most common genetic alteration in CM, reported in 15–20% of cases. Hotspot mutations in the *NRAS* gene occur mainly in exon 2 (codons 12 and 13) and exon 3 (codon 61) [31]. Current evidence reveals prominent *PI3K/AKT* signaling in *NRAS* G12 mutant cells and augmented *MAPK* signaling in *NRAS* Q61 variants, suggesting that *NRAS* G12 and *NRAS* Q61-mutant melanoma tumors differ in biology. Therefore, *NRAS*-mutant tumors might be approached with more specific and effective therapeutic strategies in the future [32]. Moreover, compared to *BRAF*-mutant tumors, *NRAS*-mutant melanomas tend to be thicker, with increased mitotic rates, usually occurring in older patients (>55 years) with a history of chronic UV exposure [33,34,35,36]. Several recent studies have also shown that *NRAS* mutations might result in inferior clinical outcomes with a lower survival rate [37]. Despite the general opinion, which assumes that *BRAF* mutations cannot coexist with *NRAS* mutations in melanocytic tumors, several research groups have also reported their synchronous occurrence [24,38]. Finally, *KRAS* is the rarest mutated proto-oncogene in melanoma (~2% of cases). *KRAS* genetic alterations also imply the alteration of the G12, G13, and Q61 residues of the protein [33,39]. It is also worth mentioning that *KRAS* mutations have been recently linked with an increased propensity of melanoma tumors to metastasize to the brain and, therefore, envisioned as valuable biomarkers of patient risk stratification in clinical settings [40].

The activation of the RAS signaling pathway by *EGFR* in cancer is well known [41]; however, in CM, there is limited information available on the role of this tyrosine kinase receptor (TKR), apart from its expression in melanocytic nevi and some melanomas [42,43,44,45]. CM is a heterogeneous disease, and its treatment is challenging; therefore, new therapeutic options are needed. As targeted anti-*EGFR* therapy has shown promising results in decreasing melanoma cell growth and hindering its invasive abilities [46], we examined melanomas and their precursors for mutations that lead to the constitutive activation of kinase activity, such as exon 19 deletions (Del19) [41]. Studies of melanoma cell lines that express *BRAF* mutation indicate a mechanism related to *EGFR* overexpression and activation of the *MAPK* and *PI3K-AKT* pathways after drug administration [47]. Moreover, melanoma cell lines with higher *EGFR* expression are more prone to becoming resistant to vemurafenib than cells with lower *EGFR* expression [48]. Additionally, melanomas treated with BRAF inhibitors tend to accumulate mutations in *NRAS* or *MEK1/2*, exhibiting excessive activation of *ERK1/2* or *SFK-STAT3* [49]. Therefore, tumor cells may again be sensitized to vemurafenib in the therapeutical approach if the drug is administered simultaneously with EGFR and AKT inhibitors [41]. Since there are very few studies investigating the mutations of this pathway in solid tumors (only 9 out of a total of 32 in the last five years), we have embarked on a pilot study on skin melanomas to assess the *RAS-RAF-MAPK* pathway mutations. Therefore, in the present study, we evaluated the *RAS*, *BRAF*, and *EGFR* mutational status of tissue specimens representing normal skin, benign nevi, and cutaneous melanomas to determine the genetic landscape of pre-malignant and malignant stages of melanoma development and to investigate the extent to which these mutational signatures overlap.

## 2. Results

### 2.1. Histopathological Evaluation of Tissue Samples

In Table 1 and Table 2, an overview of the investigated samples is presented as the demographic/clinical data of the patients.

We have studied 15 cases of acquired melanocytic nevi and their adjacent normal skin in patients from Colentina Clinical Hospital. The patients were aged 13 to 72 years (mean of 39.8), with seven men and eight women. The investigated nevi were located on the trunk (*n* = 8) and head/neck (*n* = 7), with three cases on the scalp and four cases on the face. The histological presentation was of intradermal nevus (53.33%), junctional nevus (20%), and mixed nevus (26.67%). All nevi had driver mutations in the *MAPK* pathway: 13 nevi had *BRAF* V600E mutations, and the remainder had *NRAS* mutations (1 *NRAS* Q61 and 1 *NRAS* G12/G13). *NRAS* G12/G13 mutations coexisted with *BRAF* V600 mutations in five nevi. It is also worth mentioning that *NRAS* G12/G13 mutations occurred in one of the healthy skin specimens included in our study without exhibiting the same mutation in the adjacent nevi.

We analyzed 22 cutaneous melanoma samples from Colentina Clinical Hospital. The clinical cohort consisted of 13 (59%) males and 9 (41%) females, with a median age of 64.5 years (SD = 13.10). Five of the subjects who provided melanoma tumors had previously contributed with matched specimens of melanocytic nevi and healthy skin. We found that 68.2% (*n* = 15) of the melanomas were superficial spreading melanomas (SSM), and the remaining were nodular melanomas (NM). The median Breslow index was 2.4 mm, and the median mitotic index was 3 mitoses/mm^2^. The most common site of melanoma occurrence was the trunk (59.09%), followed by the limbs (22.73%) and head and neck (18.18%). Among the cases, 86.36% were classified at Clark level IV, and 13.64% were at Clark level III. Ulceration was identified in 54.55% of cases. Tumor-infiltrating lymphocytes (TILs), both brisk and non-brisk, were present in 59% of the melanoma specimens. The perivascular invasion was noted in 27.3% of cases, and regression was observed in 77.27%. Furthermore, 22.7% of the melanoma cases occurred in association with a nevus.

Among the melanoma samples in our study, 12 exhibited *BRAF* mutations, and 11 had *NRAS* G12/G13 mutations. The coexistence of *BRAF* and *NRAS* mutations was noted in eight tumor specimens, while seven samples tested as wild-type (WT) for both *BRAF* and *NRAS* genes.

We also tested *EGFR* exon 19 (Del19 *EGFR*) deletions and *KRAS* Q61 mutations, and neither of these deregulations were found in the healthy skin, benign nevi, or melanoma samples.

### 2.2. Evaluation of BRAF Mutational Status in Nevi, Perilesional Skin, and Melanomas

**Figure 1:**
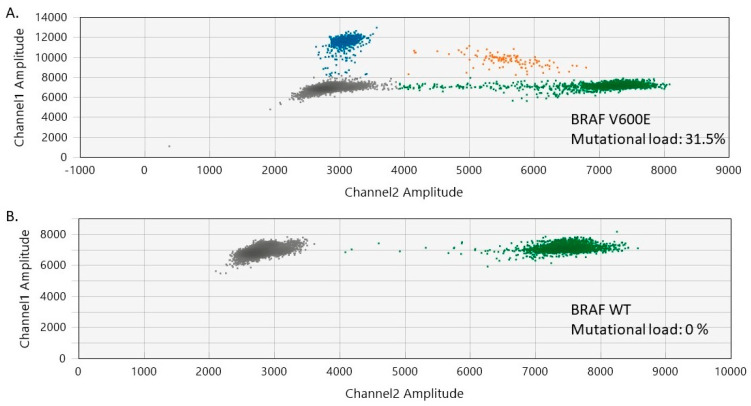
Two-dimensional ddPCR plots of *BRAF* V600 mutation detection in melanocytic nevi. (**A**) ddPCR results for a melanocytic nevus harboring *BRAF* V600 mutations (mutational load of 31.5%). (**B**) ddPCR results for a normal control DNA sample (*BRAF* V600 wild-type/WT). The green dots represent the BRAF WT-positive droplets, whereas the grey dots depict the empty/negative droplets. Mutant alleles are detectable on channel 1 (FAM labeled, blue). Double-positive droplets depicted in orange contain both mutant and BRAF WT alleles.

In our study, 85.7% of the melanocytic nevi exhibited *BRAF* V600 mutations, involving 13 cases (7 females and 6 males). The median age of the patients was 36 years (range of 13–72 years). The trunk was the most common site for *BRAF* V600-positive melanocytic nevi (*n* = 7), followed by the head and neck (*n* = 6). The histological presentation was of intradermal (*n* = 7), mixed (*n* = 3), and junctional (*n* = 3) nevi. No *BRAF* mutations were detected in the normal skin adjacent to the analyzed benign nevi. The mutational load of *BRAF* V600 in the analyzed nevi varied between 3.53% and 32% (median *BRAF* V600 mutational load = 23.5%) (Figure 1 and Figure S1, Supplementary Materials).

All melanoma specimens were tested for *BRAF* mutations, with the V600 mutations found in 12 out of 22 patients (54.6%). Four melanomas associated with benign nevi harbored *BRAF* V600 mutations akin to their matched benign counterparts. The melanocytic lesions exhibited a slightly higher, though not statistically significant, median *BRAF* V600 mutational load compared to the cutaneous tumors (23.5% versus 14.62%, *p* = 0.18).

Given the scarcity of studies on ddPCR on FFPE samples, we focused on addressing all the potential technical challenges to mitigate the risk of obtaining false-positive results. The results of the qRT-PCR analysis indicate that all *BRAF* V600 mutations identified in this study are certainly *BRAF* V600E mutations.

**Figure 2:**
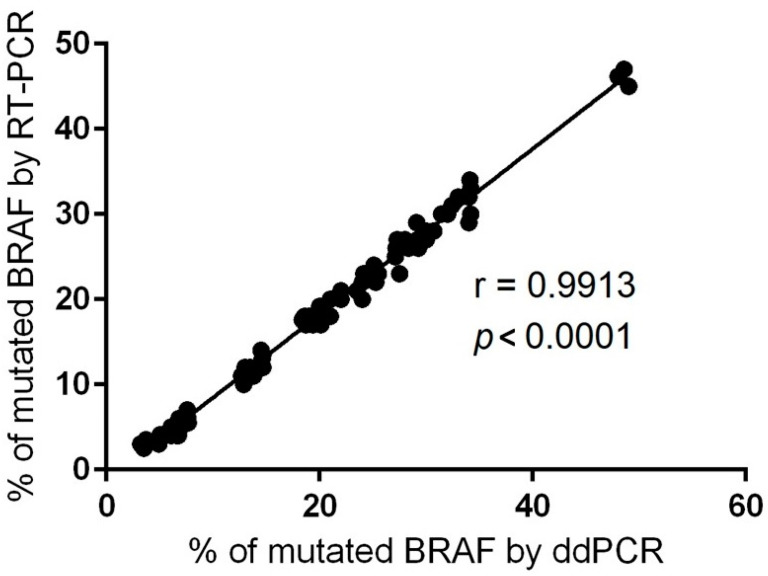
Correlation between ddPCR and RT-PCR data for *BRAF*-mutated AF% identified in the FFPE specimens (r = Spearman correlation coefficient).

As ddPCR is a relatively new technology, we performed the correlation with classical qRT-PCR regarding *BRAF* V600E mutation detection. For this purpose, we employed a CE IVD-validated RT-PCR assay with a limit of detection (LoD) of 0.05%. The Spearman’s rho test showed high conformance between the two methods on *BRAF* V600 AF% quantification (r = 0.9913, *p* < 0.0001) (Figure 2). This accordance provides direct proof of the accuracy of ddPCR technology and encourages us to use this technique further in an extended number of samples.

### 2.3. Evaluation of BRAF Mutational Status in Cutaneous Melanomas

In our batch of melanoma samples, there were 12 samples positive for *BRAF* V600E (54.6%), with mutational loads ranging from 5% to 48.6%. Further, we sought to explore potential correlations between *BRAF* mutational status and the clinicopathological features of the tumors to obtain a more comprehensive understanding of the role of this mutation in cutaneous melanoma.

We observed associations between *BRAF* V600 mutational status and Breslow thickness (*p* = 0.029), and further with the TIL score (*p* = 0.027) (Table 3). However, no associations between patient age, gender, Clark level, mitotic index, tumor localization, histological type, ulceration, and *BRAF* mutational status were found. 

Consequently, our study emphasizes a dual role of *BRAF* mutations in cutaneous melanoma—one linked with the promotion of tumor growth and another associated with modulation of the immune response. It is important to highlight that our findings align with previous observations within the field, as recently elucidated [24].

**Figure 3:**
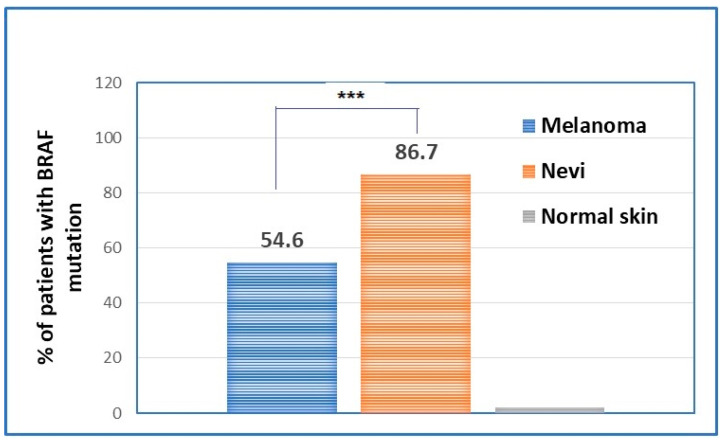
Percentage of patients that have *BRAF* mutation in the primary melanomas in comparison to the percentage of nevi that present the *BRAF* mutation. *** *p* < 0.001.

The evaluation of *BRAF* mutations in nevi and melanomas has shown statistically significant differences between the two types of samples in terms of patients’ prevalence. Hence, if the majority of nevi are *BRAF*-mutated, melanomas have only around 50% of *BRAF* mutation present (Figure 3).

### 2.4. Evaluation of NRAS Mutational Status in Nevi, Perilesional Skin, and Melanomas

Our study identified *NRAS* mutations in 7 out of 15 nevi (47.7%). Among them, six cases exhibited *NRAS* G12/G13 mutations (at a low allelic frequency ranging between 0.3% and 0.7%), and one showed an *NRAS* Q61 mutation. Two nevi harbored exclusively *NRAS* mutations, whereas the remaining five specimens demonstrated the coexistence of both *NRAS* and *BRAF* mutations. Regarding the nevi with singular *NRAS* mutations, one case with *NRAS* Q61 mutations occurred on the face of a 15-year-old male and was classified as intradermal. The mutational load of *NRAS* Q61-positive nevus was 13.47%. The other case, harboring a singular *NRAS* mutation (G12/G13), occurred on the trunk of a 33-year-old female and was classified as a mixed melanocytic nevus. Both cases can be characterized as congenital nevi, as it is known that *NRAS* mutations and not *BRAF* ones are harbored by congenital nevi [50].

**Figure 4:**
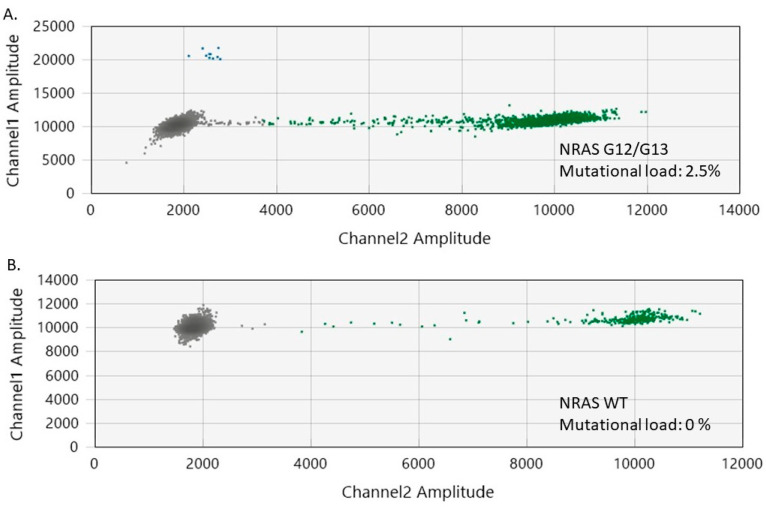
2 Two-dimensional ddPCR plots of *NRAS* G12/G13 mutation detection in melanoma samples. (**A**) ddPCR results for a melanoma sample with *NRAS* G12/G13 mutation. (**B**) ddPCR results for a melanoma sample without *NRAS* G12/G13 mutation (wild-type). The green dots represent the NRAS WT-positive droplets, whereas the grey dots depict the empty/negative droplets. Mutant NRAS G12/G13 alleles are detectable on channel 1 (FAM labeled, blue).

It is also worth mentioning that *NRAS* G12/G13 mutations occurred in one of the healthy skin specimens included in our study. Interestingly, the healthy skin sample with mutation originated from the patient that provided the benign nevi that had the *BRAF* mutation (Figure 4). *NRAS* G12/G13 mutations were recently investigated in healthy skin, with the current literature assigning a weak oncogenic role to these mutations [51,52,53].

Among the melanoma samples included in our study, 11 (50%) had *NRAS* G12/G13 mutations (mutational load ranging from 0.4% to 2.5%) (Figure 4). The coexistence of *BRAF* and *NRAS* mutations was noted in eight tumor specimens, while seven samples tested as wild-type (WT) for both *BRAF* and *NRAS* genes. Interestingly, we observed that the melanomas had a higher median *NRAS* G12/G13 mutational load than the nevi (1% versus 0.4%, *p* = 0.02).

As numerous studies have highlighted that age has a substantial effect on the acquisition and selection of cancer-driver mutations in sun-exposed normal skin [53], we aimed to assess whether this observation was valid in our batch of samples. Statistical analysis of the data demonstrated that the global somatic mutation load of tissue samples (encompassing both benign and malignant cases) exhibits no correlation with patient age (r = 0.068, *p* = 0.20), emphasizing the potential occurrence of elevated mutational rates among individuals in younger age groups.

### 2.5. Evaluation of NRAS Mutational Status in Cutaneous Melanomas

In our set of melanoma samples, encompassing 11 specimens positive for *NRAS* mutations, we performed a statistical analysis to investigate potential correlations between *NRAS* mutational status and the clinicopathological variables of the tumors.

Interestingly, *NRAS* mutational status exhibited correlations with Breslow thickness (*p* = 0.01) and the mitotic index (*p* = 0.04) (Table 4). However, we found no associations between *NRAS* mutational status and patient age, gender, Clark level of invasion, tumor site, histological type, ulceration, or presence of TILs.

Therefore, our study highlights that *NRAS* mutations may serve as indicators of disease aggressiveness in CM, particularly evidenced by parameters such as increased Breslow thickness and mitotic rate.

### 2.6. Immunohistochemical Evaluation of p16, p21, bcl2, p53, and Cyclin D1 Expression

**Figure 5:**
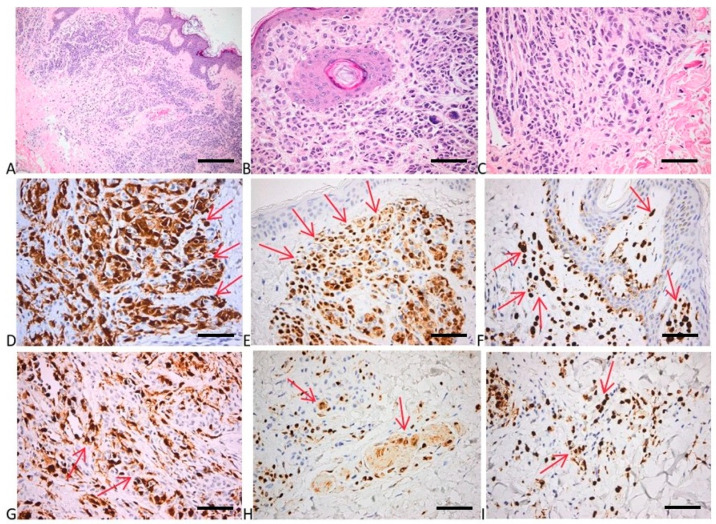
Histology and immunohistochemistry of nevus. (**A**) Papillomatous intradermal nevocellular nevus with larger nests in the superficial areas, and smaller tumor structures in the deeper parts. HE × 100 (scale bar = 200 μm). (**B**) Subepithelial areas with nests of round to cuboidal melanocytes with no nucleo-cytoplasmic atypia; a few giant multinucleated nevus cells with small nuclei laying in rosette-like arrangement. HE × 400 (scale bar = 50 μm). (**C**) Smaller and more spindly nevus cells in the deepest part of the tumor with neuroid appearance (maturation). HE × 400 (scale bar = 50 μm). (**D**–**F**) p16 positivity of the tumor cells in the upper dermis: numerous cells with strong cytoplasmic and nuclear positivity. P16 × 400 (scale bar = 50 μm). (**G**–**I**) Fewer numerous tumor cells positive for p16 in the deep dermis than in the superficial areas; preservation of both cytoplasmic and nuclear expression. P16 × 400 (scale bar = 50 μm). Red arrows depict the site of the positive expression (brown).

**Figure 6:**
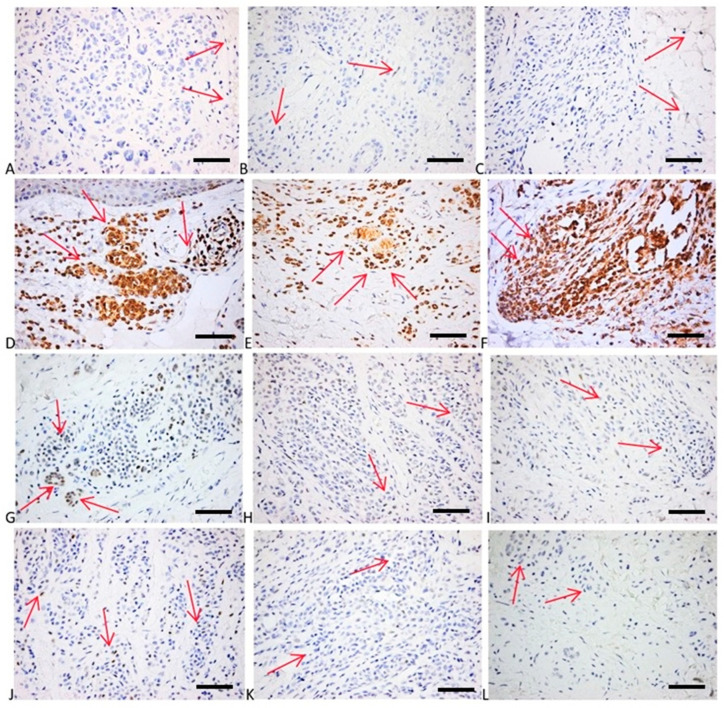
(**A**–**C**) P21 expression in melanocytic nevi: occasional tumor cells with faint nuclear positivity for p21 in both superficial (**A**) and deep part of the tumor (**B**,**C**). p21 × 400; (**D**–**F**) Bcl2 expression: strong diffuse cytoplasmic positivity in superficial tumor nests (**D**) and deep more neuroid tumor cells (**E**,**F**). bcl2 × 400; (**G**–**I**) p53 expression: admixture of negative cells and few weakly and more strongly positive cells (wild-type pattern), with a tendency for more numerous tumor cells positive in the superficial part of the tumor (**G**) than in the deeper counterparts (**H**,**I**). p53 × 400; (**J**–**L**) cyclin D1 expression: few faint positive tumor nuclei. Cyclin D1 × 400. Red arrows depict the site of the positive expression (brown) (scale bar = 50 μm).

To evaluate the proliferation, cell cycle, and apoptosis of melanocytes within the investigated samples, we evaluated p16, p21, bcl2, p53, and cyclin D1 expression in both nevi and melanoma samples. Interestingly, as the majority of the investigated nevi presented driver mutations in the MAPK signaling pathway, the expression of the p16 protein was high, proving once more that, although the melanocytes within the benign proliferation have a high mutational burden, the cell cycle of the cells are in an arrested stage (Figure 5 and Figure 6). The p16 positivity of the tumor cells in the upper dermis was high, displaying numerous cells with strong cytoplasmic and nuclear positivity. In the deep epidermis, we depicted fewer numerous tumor cells positive for p16 than in the superficial areas (Figure 5). Other investigated expressions showed that p21 had a faint nuclear positivity in the superficial and deep parts of the tumor. Bcl2 expression had a strong diffuse cytoplasmic positivity in superficial tumor nests, while p53 expression depicted a mixture of negative cells and a few weakly and more strongly positive cells (with a wild-type pattern). Cyclin D1 expression showed a few faint positive tumor nuclei (Figure 6). Negative controls of the investigated samples for p16, p21, bcl2, p53, and cyclin D1 expression are presented in Figure S2, Supplementary Materials.

Moreover, the *BRAF* and *NRAS* mutations within the nevi did not correlate with sun exposure localization, as it was equally found in sun-exposed (scalp) and non-exposed (lumbar) regions.

## 3. Discussion

Studies performed on individual healthy-skin melanocytes show that, as expected, sun-shielded melanocytes had fewer mutations related to UV signatures than sun-exposed melanocytes. In addition, melanocytes from chronically sun-exposed areas (e.g., the face, neck, and bald scalp) displayed a lower mutational burden than melanocytes on intermittently exposed skin (e.g., the back and the limbs) [12]. This assertion mirrors the clinical observations that, compared to other forms of skin cancer, melanomas are more common in intermittently exposed skin than in chronically exposed skin [54,55,56].

UVR induces erythema, edema, and dermal vasodilation, a clear portrait of inflammation events. As sunburn is a transient inflammatory response, chronic UVR contact activates prolonged inflammatory factors. Chromophores can absorb certain wavelength ranges that induce inflammation. The main chromophores for UVR in the human skin are nucleic acids, urocanic acid, amino acids with aromatic structures, and melanin [57]. The action of UVR on these chromophores initiates events that trigger inflammatory and immunological processes that favor skin tumorigenesis [58]. Furthermore, melanocytes located in the vicinity of a skin cancer were reported as having a similar mutational burden to melanomas, but a significantly higher mutational burden compared to melanocytes from donors without skin cancer, suggesting that the mutation burden of normal skin may be exploited as an indicator of UV-related DNA damage and skin cancer predisposition [12]. The genes most frequently mutated in healthy melanocytes encode for suppressors of the *MAPK* pathway, such as *NF1*, *CBL*, and *RASA2*, but gain-of-function alterations in the *BRAF*, *NRAS*, and *MAP2K1* genes have also been reported [12]. In line with these findings, we identified *NRAS* G12/G13 mutations in a healthy skin specimen included in our study. In particular, for *NRAS* G12/G13 mutations, the current literature highlights that they tend to be weakly oncogenic, giving rise to noticeable lesions only in conjunction with additional oncogenic mutations [53,59].

Melanomas often arise from typical precursor lesions, such as benign nevi, dysplastic lesions, or melanoma in situ, which allows for tracking their evolution [60,61,62]. Somatic mutations in key melanoma oncogenes, such as *BRAF* or *NRAS*, are already present in benign nevi, indicating that they occur early during disease progression. However, little is known about the additional mutations that drive the neoplastic transformation of benign nevi and their sequential order [63]. Interestingly, in our nevi samples, the majority have shown *BRAF* mutations, and some of them also *NRAS*, confirming previous studies. But although nevi are common, and they have such a high rate of mutations, they stop growing on their own [64]. The arrested growth of nevi was shown to be the trigger for oncogene-induced senescence as an intrinsic cellular response hindering proliferation [65]. However, as not all nevi have senescence markers, and some nevi can take up growing after decades or even transform into skin melanomas, a hypothesis has developed that there are also external signals that hinder nevi proliferation [66,67]. *CDKN2A* is a gene located at chromosome 9, band p21.3, and encodes p16INK4a and p14arf proteins [68], p16 being an important negative cell cycle regulator [69]. P16 is critical for melanocyte’s senescence, hindering tumorigenesis toward melanoma [70]. As we previously published, nevi have a high expression of p16 [71]. In our current work, the evaluated nevi had a high mutational burden but doubled by a high p16 expression, proving that the cellular cycle is arrested in the nevi.

According to the Clark model, which describes the histopathological changes occurring during normal melanocytes’ linear progression to melanoma via a benign naevus, *BRAF* mutations have a crucial role in melanoma development and progression [72]. *BRAF* mutations were first described in 2003 when Pollock and colleagues discovered them in 80% of their batch of nevi samples [73]. Further studies aiming to assess the mutational rate of *BRAF* in acquired melanocytic lesions have demonstrated similar prevalence rates; however, *NRAS* mutations were identified at a much lower percentage (6%) in benign melanocytic nevi [74]. Moreover, Shain et al. reported that *NRAS* and *BRAF* mutations may have a strict specificity regarding the type of nevi in which they occur, with *BRAF* V600E mutations being more frequent in benign lesions compared to *NRAS* mutations, which occur predominantly in intermediate precursor lesions, such as dysplastic nevi [75]. Despite the claimed oncogenic role of *BRAF* mutations, the mutant melanocytes within a benign nevus lose their proliferative activity, as the entire benign entity comprising them stops growing and temporarily stabilizes in size. These observations are supported by the fact that *BRAF* V600E mutations can also be present in normal human melanocytes, triggering cell cycle arrest and p16INK4a overexpression, features also reported in early-stage melanomas [70,76]. Thus, acquired melanocytic nevi are likely benign clonal tumors that are initially stimulated to proliferate via oncogenic *BRAF* signaling but which finally undergo growth arrest due to oncogene-induced senescence [74]. It is unanimously accepted that *BRAF* gene mutations are insufficient to drive the malignant transformation of normal melanocytes; thus, more research regarding the factors involved in this process is needed [16,77]. Finally, the almost perfect overlap of the *BRAF* mutational profile between melanomas and their benign counterparts, together with the existence of some *BRAF* wild-type melanomas originating from *BRAF*-mutant nevi, suggests that melanomagenesis is a multifaceted process that involves numerous factors, many of them not yet elucidated [78]. Here, we report in 85.7% of the cases the presence of *BRAF* V600E mutations for the analyzed benign nevi. Moreover, we highlight that *NRAS* G12/G13 mutations may also be present but at very low allelic frequencies in acquired melanocytic nevi. Usually, there is no overlap regarding mutations in both *NRAS* and *BRAF* in nevi or CM [16], but surprisingly, we reported several cases of simultaneous mutations in both of those two genes. We consider that the detection of *NRAS* G12/G13 mutations was enabled by the ultrasensitive power of ddPCR, which has a limit of detection (LoD) of 0.005%. The unique *NRAS* mutation obtained in the two cases of benign nevi sustains the fact that the investigated nevi were congenital. However, the precise role of *NRAS* G12/G13 mutations in benign nevi is not yet well defined and remains to be explored in further studies. At least in our batch of investigated nevi, the *NRAS* mutations accompanying *BRAF* mutations were not associated with the nevi location, highly exposed versus non-exposed skin areas, as were detected on the scalp or trunk.

As mentioned, the most frequently mutated genes in cutaneous melanoma are *BRAF* and *NRAS*. The *BRAF* V600 mutation has been linked to younger age at diagnosis, lack of chronic UV damage, a high total body nevus count, and localization of the primary tumor on the body extremities [25,26]. Some studies also suggest an association with the male gender [79] or any gender association [80]. The frequency of *BRAF* mutations in primary melanomas is commonly reported to be between 40 and 60% [21,22], with 54.6% seen in our study. Regarding tumor characteristics, at least three studies demonstrated an association with tumor thickness [24,36]. Furthermore, other investigations suggest a correlation between *BRAF* mutation and aggressive tumor features, such as increased mitotic rates [81] and the presence of ulceration [82,83]. However, our study found no associations between the patient age, gender, Clark level, mitotic index, tumor localization, histological type, ulceration, and the *BRAF* V600 mutational status. The only significant associations were with Breslow thickness (*p* = 0.029) and the presence of TILs (*p* = 0.027). Thus, our study highlights a dual role of *BRAF* V600 mutations in cutaneous melanoma: one related to sustaining tumor growth and another linked to the modulation of the immune response.

In accordance with previous evidence [84,85] and our findings, a potential correlation is emerging between the presence of TILs and *BRAF* mutation in CM. The current literature also highlights that half of the primary *BRAF*-mutated melanomas exhibit the non-brisk phenotype, while approximately 30% fall within the TIL brisk group [86]. However, while the clinical significance of TILs in *BRAF*-mutant melanomas remains largely unknown, the immunomodulatory effects linked to *BRAF*-targeted therapy–leading to increased expression of melanoma antigens and a more favorable tumor microenvironment—indicate that this feature may hold particular importance in the therapeutic setting [87]. Therefore, adopting TIL assessment in routine histopathological practice may be helpful for melanoma risk stratification and therapeutic guidance.

Mutated *NRAS* is the second most frequent genetic alteration in CM, occurring in 15% to 20% of cases [31]. In our study, the frequency of *NRAS* mutations was higher than previously reported (50%), and we attribute this to the ultrasensitive power of the ddPCR technology. It has been demonstrated that patients with *NRAS* mutant melanoma were older than 55 years and had a previous history of UV exposure, and thus, higher pro-inflammatory conditions than those with *BRAF* mutant tumors [31]. *NRAS*-mutant melanomas are typically located on the upper extremities [37]. In addition, *NRAS* mutations were also found to be associated with the nodular subtype of the primary tumor [13] and with unfavorable prognostic factors, such as increased Breslow thickness, mitotic activity, and lymphovascular invasion [35,36,73].

In our small batch of melanoma samples, *NRAS* mutational status exhibited correlations with two of the most important histological predictors of clinical outcome: Breslow thickness (*p* = 0.01) and the mitotic index (*p* = 0.04). We observed no associations between *NRAS* mutations and the patient age, gender, Clark level of invasion, tumor site, histological type, ulceration, or presence of TILs. Therefore, our study highlights the potential involvement of *NRAS* mutations in modulating the aggressiveness of CM. These findings align with previous studies in the field [24,33,36] and underscore the importance of *NRAS* mutation molecular testing in clinical practice. Such testing may offer insights into tumor behavior and significantly contribute to patients’ risk stratification.

*NRAS* mutations are known to occur independently of *BRAF* mutations in melanocytic tumors; however, recent research using advanced technologies revealed that these two mutations may co-exist in almost one-third of CM cases. Patients with both *BRAF* and *NRAS* mutations have a poorer prognosis compared to those bearing either *NRAS* wild-type or *BRAF*-mutant melanoma [37]. Our study confirms the co-occurrence of *BRAF* and *NRAS* mutations in melanoma and emphasizes the metastatic potential of a melanoma tumor that displays both of the mentioned mutations. Moreover, we show the co-occurrence of *BRAF* and *NRAS* mutations for the first time even in acquired melanocytic nevi.

At the same time, we did not find any *EGFR* exon 19 (Del19 EGFR) deletions or *KRAS* Q61 mutations in the healthy skin, benign nevi, or melanoma FFPE specimens. *EGFR* exon19 deletion characterizes non-small cell lung cancer and confers sensitivity to EGFR tyrosine kinase inhibitors (TKIs) [88]. No reports were found regarding this deletion in melanomas. Few reports show *KRAS* Q61 mutations in UV-inducible melanoma models [89]. Using the same ddPCR technology (Bio-Rad Laboratories, Hercules, CA, USA), an *NRAS* Q61 mutation was found in malignant peripheral nerve sheath tumors that were further identified as melanoma [90].

However, it is still unknown for CM if the oncogenic *BRAF* gene is capable of maintaining oncogenic *MAPK* kinase signaling on its own.

The intensely debated role of *BRAF* mutations in benign nevi outlines two distinct hypotheses regarding their functionality in benign and pre-malignant settings. Despite the notable changes reported in melanocytic nevi after exposure to BRAF inhibitor treatment, a significant percentage of nevi do not regress when exposed to targeted therapies such as vemurafenib, although they are underpinned by oncogenic *BRAF* V600E mutations [91]. This lack of sensitivity to therapy suggests that within the melanocytic nevi genome, there may be an internal inhibitor that attenuates the pro-oncogenic activity associated with *MAPK* signaling, preventing nevi from behaving like a malignant tumor and regressing under targeted therapy (the tumor-suppressor hypothesis). In parallel, another hypothesis (the second oncogene hypothesis) claims that not the *BRAF* gene but several cofactors that acquire oncogenic mutations are the key players that facilitate the neoplastic transformation of melanocytic nevi into CM [91]. Therefore, additional studies are needed to unravel the genomic landscape of healthy skin and benign melanocytic nevi and determine the key genetic alterations triggering melanocyte neoplastic transformation.

Using The Cancer Genome Atlas (TCGA) for melanoma, it was shown that in all stages, there are genes significantly related to *MAPK*, neurotrophin, the focal adhesion signaling pathway, and, moreover, immune and inflammation pathways. Recently, a six-lncRNA prognostic signature that can stratify risk patients was reported in relation to these genes [92]. Although *MAPK* pathway mutations can induce the proliferation of melanocytes within a nevus, it is obvious that not all nevi (bearing *BRAF* mutations) will turn to melanoma. Thus, our results come in favor of the oncogene-induced senescence hypothesis [64], as oncogene activation induces growth arrest.

As a limitation, our study has a low number of cases. We compensated for this by evaluating a high number of genetic parameters performing a thorough IHC assessment, with the results confirmed by recent publications as well. ddPCR technology has a high sensitivity [93] and can detect lower levels of mutations in the FFPE tissue samples, therefore recommending this technology for further extended studies. Another limitation of our study is that mutational screening is performed on a diverse collection of skin cells, which generates an average signal of the tissue sample instead of depicting the mutational landscape of individual melanocytes. Moreover, although we report the coexistence of *BRAF* and *NRAS* mutations within the same tumor, this does not necessarily imply these mutations can co-occur within single cells, as each tumor cell has its transcriptional repertoire that dictates its fate. Thus, although the present study offers a comprehensive overview of the mutational landscape of healthy skin, nevi, and cutaneous melanomas, the most accurate information regarding the molecular events that lead to melanoma can be obtained through single-cell sequencing. While single-cell sequencing technologies are expensive and require sophisticated technology and trained personnel, they have the potential to provide a more exhaustive picture of CM intratumoral heterogeneity and the associated transcriptional programs that modulate disease progression, drug sensitivity, or tolerance, holding promise for more personalized approaches in the clinical setting.

**Figure 7:**
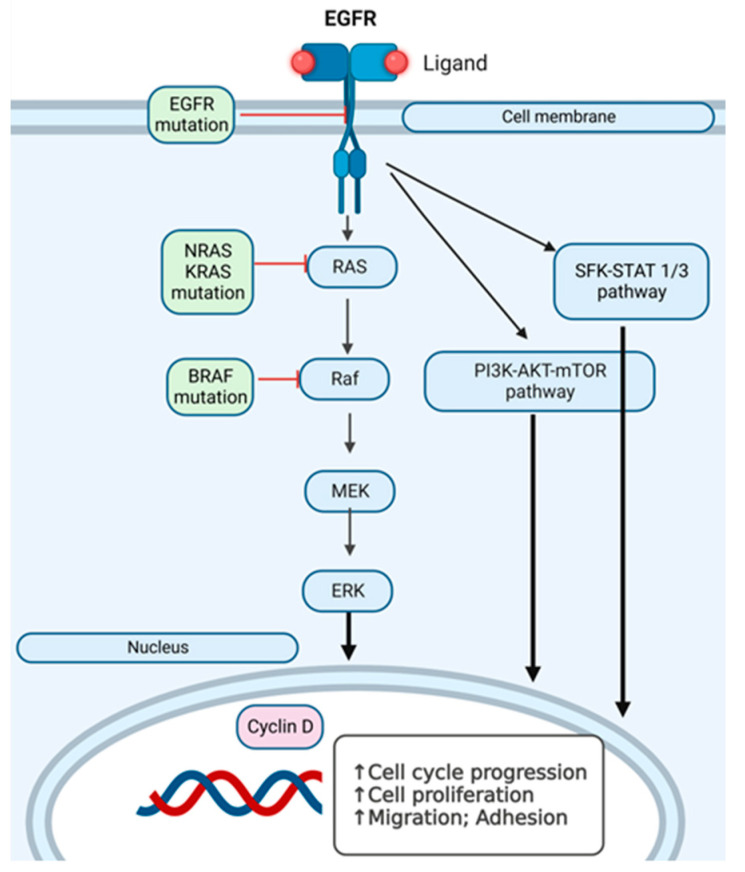
Intracellular signaling pathways in *RAS-RAF-MAPK* activation in melanoma cells. *SFK-STAT1/3* (Src family kinase-signal transducer and activator of transcription 3) pathway, *PI3K-AKT-mTOR* pathway and *RAS-RAF-MEK* pathway, which can have several points where mutations can appear. The arrows (↑) indicate the stimulation of the indicated cellular processes.

We have investigated *EGFR* and *RAF-RAS-MAPK* pathway mutations in the search for additional hit targets for further therapeutical approaches that can overcome immunotherapy and/or targeted therapy resistance, with the aim of personalizing the therapy. In Figure 7, we present an overview of the pathways we have investigated at the mutational level in skin melanomas, nevi, and healthy skin.

Future perspectives of this complex genetic evaluation that are still in the research stages can drive new therapeutical approaches for CM, such as one-time autologous TIL cell therapy [94], extracellular vesicles as drug transporters [95], or new adjuvants like peptides [96,97].

## 4. Materials and Methods

### 4.1. Patients

The present study aimed to assess the roles and prevalence of *RAS-RAF-MAPK* signaling pathway mutations in melanoma tumors, benign moles, and normal skin samples via the ddPCR and immunohistochemistry (IHC) techniques. Twenty-two melanoma specimens were obtained from patients with a clinical history of melanoma who had not undergone neoadjuvant treatment before surgical resection of the lesions. Fifteen acquired melanocytic nevi, along with corresponding adjacent normal skin (perilesional), were excised from 15 patients with (*n* = 5) or without a history (*n* = 10) of skin melanoma. All individuals that comprised the presented groups were phototype 2, with ages ranging from 13 to 87 years. Clinical and demographic characteristics of the patients are presented and detailed in Table 1 and Table 2 in Section 2.

This study was conducted in accordance with the principles of the Declaration of Helsinki (according to Annex 4, National Law 104/2004 [98] and application HG451/2004, amended in 2011 [99]) and with the approval of the Local Ethics Committee of Colentina Clinical Hospital, Bucharest, Romania (approval no. 25/2017). All patients and control subjects were informed of the study protocol and provided written informed consent before enrollment. Their personal data were kept confidential during this study, and only their physicians (dermatologists and pathologists) could access their identity.

### 4.2. Histopathological Evaluation of Tumor Samples

The histopathological characteristics of melanoma and nevi were assessed by two expert pathologists (LN and CP, authors of the present study) according to the latest American Joint Committee on Cancer (AJCC) tumor/node/metastasis (TNM) classification and staging system. Clinicopathological features, such as the tumor type, Clark invasion level, Breslow thickness, mitotic activity, ulceration, lymphovascular invasion, tumor-infiltrating lymphocytes (TILs), and regression, were recorded for each sample. The primary method employed to evaluate the histopathological modifications and TILs in the tissue specimens was H&E staining.

Samples were obtained by the Pathology Department of Colentina Hospital as fresh or formalin-fixed tissue. During the macroscopic examination (grossing), all the fragments were put in cassettes and immersed in 10% buffered formalin until the next day (18–24 h). Further routine histopathological processing to paraffin with the automatic tissue processor Leica Peloris 3 was performed. The tissue was embedded in paraffin blocks (using embedding stations Thermo Fisher Microm EC 1150 H (Waltham, MA, USA), Leica EG 1150H (Wetzlar, Germany), Sakura Tissue Tek (Torrance, CA, USA) and Leica Arcadia), sectioned at 3 microns thick (semi-automated Rotary Microtome Leica RM2255 and RM2265), and stained with hematoxylin–eosin (H&E) on the Leica Spectra staining station. The first section was stained with H&E, and the subsequent ones were evaluated for their expression of cell cycle regulators and immune markers by standard immunohistochemistry (IHC). H&E staining was performed according to the standard procedure implemented in the Colentina Hospital hosting the patients’ samples [100].

### 4.3. Assessment of the Inflammatory Infiltrate of Cutaneous Tumors

The histopathological evaluation of TILs was performed through a semi-quantitative method employing light microscopy on the H&E-stained melanoma tissue sections (magnification ×100–400). TIL assessment was conducted by two experienced pathologists according to Clark’s scoring system [101] and the latest version of the College of American Pathologists (CAP) Protocol for the examination of biopsy specimens from patients with melanoma of the skin (version: 4.3.1.0, updated in March 2022) [102]. According to Clark’s TIL scoring system, to qualify as TILs, lymphocytes must surround and disrupt tumor cells in the vertical growth phase. Based on their distribution and density patterns, TILs are classified as “brisk”, “non-brisk”, or “absent”. Lymphocytes are described as “brisk” if they diffusely infiltrate tumor edges or the entire invasive component of melanoma. In contrast, lymphocytes are graded as “non-brisk” if they penetrate the melanoma only focally, without affecting the whole base of the invasive tumor. The TIL grade may also be categorized as “absent” if lymphocytes are absent or they are present but do not infiltrate the tumor mass [101] (Figure S3, Supplementary Materials).

An examination of tissue slides stained with lymphocyte markers was also conducted to confirm the presence of an immune-inflamed tumor microenvironment or histological regression. CD3, CD4, CD8 (T cells), and CD20 (B cells) expression was studied using standard IHC. Tissue slides stained with lymphocyte markers were also evaluated in accordance with the Clark grading system (Figure S4, Supplementary Materials). The Novolink Polymer Detection System (Leica Biosystems, Wetzlar, Germany) was utilized for IHC analysis of TIL subsets. FFPE tissue sections underwent deparaffinization, antigen retrieval (with EDTA or citrate buffers), and unspecific site blocking. Afterward, the slides were incubated for 1 h with primary antibodies (Table 5). Bound primary antibodies were detected after incubation with the secondary antibodies included in the Novolink Polymer Detection kit and with the diaminobenzidine (DAB) chromogen (Leica, Wetzlar, Germany). Additional details of the IHC procedure have been described elsewhere in more detail [103].

### 4.4. Immunohistochemical Analysis of p16, p21, bcl2, p53, and Cyclin D1 Expression

Standard immunohistochemistry (IHC) evaluation was performed for all the samples regarding the expression of p16, p21, bcl2, p53, and cyclin D1, along with the negative controls (Figure S2, Supplementary Materials).

Table 6 contains all the information regarding the primary antibodies and antigen retrieval procedures employed to assess cell cycle protein expression. Secondary antibodies for IHC staining were provided by the Novolink Polymer Detection kit along with the DAB chromogen (Leica) [103].

### 4.5. Genomic DNA (gDNA) Isolation from FFPE Tissue Samples

Additionally, paraffined-included tissue samples of 3 × 15 μm sections were analyzed using ddPCR technology for gene mutations in *KRAS* Q61, *NRAS* G12/G13, *NRAS* Q61, *BRAF* V600, and for *EGFR* exon 19 deletions.

Before proceeding to genomic DNA (gDNA) extraction, the FFPE tissue samples were sectioned using a manual microtome (Leica) to generate 3 sections, each with a thickness of 15 μm. During the sectioning of the FFPE blocks, all the necessary measures were taken to prevent cross-contamination or ambient contamination of the samples. Genomic DNA was isolated from the FFPE tissue sections using the ReliaPrep™ FFPE gDNA Miniprep System (#A2352, Promega, Madison, WI, USA). The average DNA concentration was 95 ng/μL (8.5 to 543 ng/μL), and the average 260/280 was 1.83.

### 4.6. Uracil-DNA Glycosylase (UDG) Treatment of FFPE gDNA Samples

Prior to ddPCR analysis, the gDNA samples were treated with uracil-DNA-glycosylase (UDG; #1B1634, VWR, Radnor, PA, USA) as per the manufacturer’s instructions to eliminate any potential C > T transitions that may occur as a consequence of tissue fixation and reduce the false-positive rate of the ddPCR assay. Then, 10 μL of gDNA was retrieved from each replicate of the analyzed samples and mixed in independent tubes with 2 μL 10× UDG reaction buffer and 2 units of UDG. The gDNA FFPE wild-type (WT) controls were treated similarly. The gDNA WT controls and experimental samples were incubated at 37 °C for 20 min.

### 4.7. Restriction Digestion of the gDNA Samples Prior to ddPCR

The gDNA samples analyzed for *BRAF* V600, *NRAS* G12/G13, *NRAS* Q61, *KRAS* Q61, and *EGFR* exon 19 deletions were digested 2–3 h at 37 °C with the following recommended restriction enzymes: Hind III, Hae III, MseI, and EcoR I (15 U/μg gDNA).

### 4.8. ddPCR Reaction Setup

The mutational status of the healthy skin, nevi, and melanoma specimens was assessed with ddPCR using *BRAF* V600 (#12001037), *NRAS* G12/G13 (#12001627), *NRAS* Q61 (#12001006), *KRAS* Q61 (#12001626), and *EGFR* exon 19 deletion (#12002392) Screening Assays (all Bio-Rad, Hercules, CA, USA) according to the manufacturer’s instructions. The primer’s design details can be found in Table S1 in the Supplementary Materials. In addition, the *BRAF* V600-positive samples were subjected to *BRAF* V600E mutation analysis by real-time PCR using the CE-IVD marked gb ONCO *BRAF* (V600E) mutation detection assay (#3241-048, GENERI BIOTECH, Hradec Kralove, Czech Republic). Droplets were generated using the Biorad QX200 Droplet Generator and analyzed with a QX200 Droplet Reader (Bio-Rad, Hercules, CA, USA). All restricted gDNA samples were tested in three replicates. Each ddPCR reaction mixture consisted of 10 μL of 2× ddPCR Supermix for Probes (no dUTP; Bio-Rad, Hercules, CA, USA), 1 μL of 20× *BRAF* (*NRAS/KRAS/EGFR*) Screening Assay (all Bio-Rad, Hercules, CA, USA), and 3 µL of gDNA template (8 ng/μL), into a final reaction volume of 20 µL. Individual ddPCR reactions were brought to a final volume of 20 µL with RNase-/DNase-free water (#10977-035, Invitrogen, Waltham, MA, USA). Each run included a negative control (NTC) and a positive control at the same concentration as the restricted gDNA samples. Positive mutation references were from Horizon Discovery (#HD701, Horizon Discovery, Cambridge, UK), presenting key mutations in the *BRAF*, *KRAS*, *NRAS*, and *EGFR* genes at allelic frequencies between 1 and 24.5%, and the negative controls were wild-type (WT)—only gDNAs from Promega Corporation (#G1471, Promega Corporation, Madison, WI, USA). All reaction mixtures were pipetted into disposable DG8 cartridges (Bio-Rad, Hercules, CA, USA) in the presence of 70 μL of droplet generation oil (Bio-Rad, Hercules, CA, USA) and partitioned into up to 20,000 nanoliter-sized droplets using the QX200 droplet generator (Bio-Rad, Hercules, CA, USA). The droplets were subsequently transferred into a 96-well plate (Bio-Rad, Hercules, CA, USA) and amplified on a C1000 Touch Thermal Cycler (Bio-Rad, Hercules, CA, USA), adopting the following thermal cycling profiles: 95 °C for 10 min, followed by 40 cycles of 94 °C for 30 s, 55 °C for 1 min, 1 cycle at 98 °C for 10 min, and ending at 8 °C. Cycling between the temperatures was set to a ramp rate of 2 °C/s, according to the manufacturer’s recommendations. Upon completion of the PCR protocol, the plate was read using the QX200 droplet reader (Bio-Rad, Hercules, CA, USA), selecting the absolute quantification of the target DNA (target copies/µL of reaction). The ddPCR data were analyzed with the analytical software QuantaSoftTM Analysis Pro (v1.0.596, Bio-Rad, Hercules, CA, USA) after setting a threshold using the fluorescence of negative controls. Each ddPCR assay employed two probes: one that emitted a FAM fluorescence signal in the presence of the mutated target and a second that emitted a HEX fluorescence signal in the presence of the wild-type (WT) target. Wells with a low number (<10,000) of total droplets were not taken into consideration. A ddPCR reaction was considered positive if at least three droplets out of ~20,000 presented FAM fluorescence. A sample was considered positive if all replicate reactions were positive. In the case of mutated samples, the mutation allele frequency (AF%) was calculated using the mutant allele concentration in copies/µL (Mut) and the wild-type allele concentration in copies/µL (WT) as follows:

AF% = Mut/(Mut + Wt) × 100

### 4.9. Statistical Analyses

All statistical analyses were performed using GraphPad PRISM v9 software (Boston, MA, USA, www.graphpad.com, accessed on 11 December 2023). We conducted descriptive statistics on the demographic and clinicopathological data of the enrolled patients, presenting absolute and relative frequencies for categorical variables and reporting the mean with the standard deviation or median with the interquartile range for numerical variables. A comparison of the mutational load between melanomas and nevi, separately for *BRAF* and *NRAS* genes, was conducted using the Mann–Whitney test. Spearman’s rank correlation coefficient was used to identify the correlation between patient age and the somatic mutation load in tissue specimens. The associations between the *BRAF/NRAS* mutation status and the clinicopathologic parameters were investigated using the Fisher’s exact test for categorical variables, and the Mann–Whitney test for continuous variables. The comparison of the ddPCR and RT-PCR methods for *BRAF* V600 allele frequency (AF%) quantification was conducted using Spearman’s rank correlation coefficient analysis. A *p*-value < 0.05 (two-tailed) was considered statistically significant.

## 5. Conclusions

Here, we employed ddPCR technology and standard IHC to assess the frequency and roles of *RAS-RAF-MAPK* pathway mutations in a group of FFPE samples harvested from melanoma patients and individuals with benign nevi.

As *BRAF* V600E testing is paramount in routine clinical testing in cutaneous melanoma, we aimed to compare ddPCR sensitivity with a CE-IVD validated RT-PCR assay when screening for these mutations in paraffin-embedded tissue samples. Interestingly, we demonstrated that ddPCR is as reliable as a CE IVD-validated technology in detecting *BRAF* V600E mutations in tissue biopsies, which can be a solid argument supporting its implementation in the clinical setting. In addition, we did not find *EGFR*-related mutations in the analyzed samples.

However, we were able to detect low-frequency hotspot mutations, such as *NRAS* G12/G13, in our healthy skin, nevi, and melanoma sample specimens, suggesting that, due to its increased sensitivity, ddPCR technology can provide new and relevant information for future studies in the field. Although the roles of *NRAS* G12/G13 mutations in premalignant and malignant settings in CM are largely unknown, we consider that by harnessing the ultrasensitive power of ddPCR, our study provides some updates regarding the molecular alterations present within sun-damaged skin, benign moles, and cutaneous melanomas. However, additional studies are needed to unravel the genomic landscape of healthy skin and benign melanocytic nevi and determine the key genetic alterations driving melanocyte neoplastic transformation.

## Figures and Tables

**Table 1:** Demographic and histopathological data of nevi samples.

	Total (*n* = 15)	
	*n*	%
Age (years)		
Mean ± SD	39.8 ± 18.32	
Median [Q1–Q3]	34 [25; 55]	
Gender		
Male	7	(46.67%)
Female	8	(53.33%)
Site		
Trunk	8	(53.33%)
Head and neck	7	(46.67%)
Nevus subtype		
Intradermal	8	(53.33%)
Junctional	3	(20.00%)
Mixed	4	(26.67%)
*BRAF* mutation		
*BRAF* V600	13	(86.66%)
*NRAS* mutation		
*NRAS* Q61	1	(6.67%)
*NRAS* G12/G13	6	(40.00%)
*BRAF/NRAS* co-mutant	5	(33.3%)

**Table 2:** Demographic and histopathological data of melanoma samples.

	Total (*n* = 22)	
	*n*	%
Age (years)		
Mean ± SD	64.5 ± 13.10	
Median [Q1–Q3]	65 [58; 72]	
Gender		
Male	13	(59%)
Female	9	(41%)
Site		
Trunk	13	(59.09%)
Head and neck	4	(18.18%)
Upper limbs	5	(22.73%)
Histological type		
SSM	15	(68.2%)
NM	7	(31.8%)
Clark level		
III	3	(13.64%)
IV	19	(86.36%)
Breslow index (mm)		
Mean ± SD	2.82 ± 2.07	
Median [Q1–Q3]	2.4 [1.3; 3.6]	
<2	10	(45.46%)
2–4	8	(36.36%)
4+	4	(18.18%)
Mitotic index (mitoses/mm^2^)		
Mean ± SD	5.63 ± 7.42	
Median [Q1–Q3]	3 [0; 6]	
0–1	7	(31.8%)
1+	15	(68.2%)
Ulceration		
Yes	12	(54.55%)
No	10	(45.45%)
TIL score		
Non-brisk	5	(22.73%)
Brisk	8	(36.36%)
Absent	9	(40.91%)
Regression		
Absent	17	(77.27%)
Present	5	(22.73%)
Perivascular invasion	6	(27.3%)
Nevus-associated melanoma	5	(22.73%)
*BRAF* mutation	12	(54.55%)
*NRAS* mutation	11	(50.00%)
*BRAF/NRAS* co-mutant	8	(36.36%)
*BRAF/NRAS* WT	7	(31.8%)

SSM: superficial spreading melanoma; NM: nodular melanoma; TIL: tumor-infiltrating lymphocytes; WT: wild-type.

**Table 3:** Statistical analysis of potential correlations between *BRAF* V600 mutational status and clinicopathological parameters.

*BRAF* Mutation				
Variable		*BRAF* V600	*BRAF* WT	*p* Value
	Number (%)	12 (54.55%)	10 (45.45%)	
Age (years)				
	Median (range)	63 (41–83)	68.5 (44–87)	0.138 ^a^
Gender				
	Male	5 (41.67%)	8 (80%)	0.099 ^b^
	Female	7 (58.33%)	2 (20%)	
Breslow (mm)				
	Median (range)	3.35 (1.1–7.5)	1.5 (0–7)	0.029 ^a,^*
Clark level				
	III	0 (0%)	3 (30%)	0.07 ^b^
	IV	12 (100%)	7 (70%)	
Mitotic index				
	Median (range)	3.5 (1–15)	0.1 (0–25)	0.11 ^a^
Site				
	Trunk	7 (58.34%)	6 (60%)	0.34 ^b^
	Limbs	4 (33.33%)	1 (10%)	
	Head and neck	1 (8.33%)	3 (30%)	
Histological type				
	NM	3 (25%)	4 (40%)	0.65 ^b^
	SSM	9 (75%)	6 (60%)	
TILs ^¶^				
	Present	10 (83.33%)	3 (30%)	0.027 ^b,^*
	Absent	2 (16.67%)	7 (70%)	
Ulceration				
	Yes	10 (83.33%)	4 (40%)	0.07 ^b^
	No	2 (16.67%)	6 (60%)	

SSM: superficial spreading melanoma; NM: nodular melanoma; ^a^ Mann–Whitney U test; ^b^ Fisher’s exact test; ^¶^ TIL grade was categorized as present (brisk or non-brisk) or absent; * *p* < 0.05.

**Table 4:** Statistical analysis of potential correlations between *NRAS* mutational status and clinicopathological parameters.

*NRAS* Mutation				
Variable		*NRAS* G12/G13	*NRAS* WT	*p* Value
	Number (%)	11 (50%)	11 (50%)	
Age (years)				
	Median (range)	68 (42–83)	63 (41–87)	0.35 ^a^
Gender				
	Male	6 (54.55%)	7 (63.64%)	1.00 ^b^
	Female	5 (45.45%)	4 (36.36%)	
Breslow (mm)				
	Median (range)	3.6 (1.7–7.5)	1.3 (0–6.25)	0.01 ^a^*
Clark level				
	III	0 (0%)	3 (27.27%)	0.21 ^b^
	IV	11 (100%)	8 (72.73%)	
Mitotic index				
	Median (range)	4 (1–25)	0.1 (0–15)	0.04 ^a^*
Site				
	Trunk	7 (63.64%)	6 (54.55%)	0.85 ^b^
	Limbs	2 (18.18%)	3 (27.27%)	
	Head and neck	2 (18.18%)	2 (18.18%)	
Histological type				
	NM	2 (18.18%)	5 (45.45%)	0.36 ^b^
	SSM	9 (81.82%)	6 (54.55%)	
TILs ^¶^				
	Present	8 (72.73%)	5 (45.45%)	0.38 ^b^
	Absent	3 (27.27%)	6 (54.55%)	
Ulceration				
	Yes	6 (54.55%)	6 (54.55%)	1.00 ^b^
	No	5 (45.45%)	5 (45.45%)	

SSM: superficial spreading melanoma; NM: nodular melanoma; ^a^ Mann–Whitney U test; ^b^ Fisher’s exact test; ^¶^ TIL grade was categorized as present (brisk or non-brisk) or absent; * *p* < 0.05.

**Table 5:** The primary antibodies used for IHC analysis of TIL subsets.

Antibody (Mouse)	Clone	Company	Working Dilution	Pre-Treatment
CD3	LN10	Leica	RTU *	HIER *, buffer citrate, and pH 6
CD4	4B12	Leica	1:100	HIER, EDTA, and pH 9
CD8	4B11	Leica	1:50	HIER, EDTA, and pH 9
CD20	L26	Leica	1:150	HIER, buffer citrate, and pH 6

* RTU—ready-to-use, HIER—Heat-induced epitope retrieval.

**Table 6:** Technical details regarding the primary antibodies and the recommended IHC pretreatments.

Antibody (Mouse)	Clone	Company	Working Dilution	Pre-Treatment
P16	6H12	Leica	RTU	HIER, EDTA and pH 9
BCL2	BCL-2/100D5	Leica	RTU	HIER, EDTA and pH 9
P21	4D10	Leica	1:20	HIER, buffer citrate and pH 6
CycD1	EP12	Leica	RTU	HIER, EDTA and pH 9
P53	DO7	Leica	RTU	HIER, EDTA and pH 9

## Data Availability

The data presented in this study are available on request from the corresponding author. The data are not publicly available due to reasons related to patient privacy and confidentiality.

## Supplementary Materials

The following supporting information can be downloaded at: https://www.mdpi.com/article/10.3390/ijms25042308/s1.
