# Supplementary material for: Assessment of RAS-RAF-MAPK Pathway Mutation Status in Healthy Skin, Benign Nevi, and Cutaneous Melanomas: Pilot Study Using Droplet Digital PCR
Source: Int J Mol Sci. 2024 Feb 15;25(4):2308. doi: 10.3390/ijms25042308 (PMC10889428; doi:10.3390/ijms25042308)
Supplement: Supplementary file 1 [file ijms-25-02308-s001.zip › ijms-2855289-supplementary.pdf]

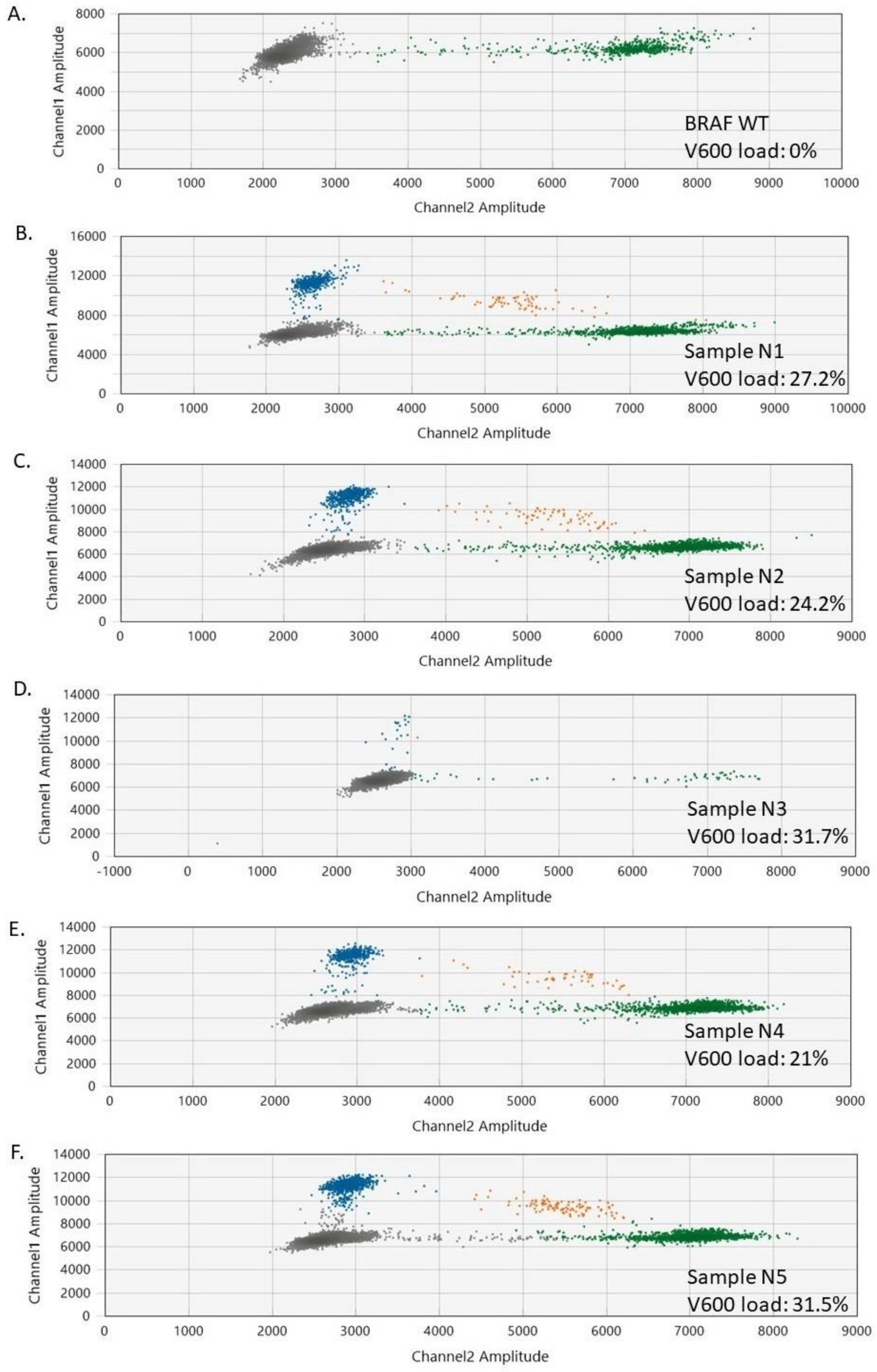

Figure S1. 2D ddPCR plots of BRAF V600 mutation detection in melanocytic nevi. (A) ddPCR results for a normal control DNA sample (BRAF V600 wild-type). (B-F) ddPCR results for 5 melanocytic nevi with BRAF V600 mutations. The BRAF V600 mutational load in the depicted nevi ranges from 21% for the N4 sample to 31.5% for the N5 sample.

Table S1. Primers designs within the ddPCR kits used for mutation detections

Assay kit name code	Mutation detected, species	Available at
ddPCR BRAF V600 Screening Kit #12001037	ddPCR™ Mutation Assay: BRAF p.V600E c.1799T>A, Human, Homo sapiens	https://www.bio-rad.com/digital-assays/assay-detail/dHsaMDV2010027, accessed on 4 December 2023
	ddPCR™ Mutation Assay: BRAF p.V600K, Human, Homo sapiens	https://www.bio-rad.com/digital-assays/assay-detail/dHsaMDV2010035, accessed on 4 December 2023
	ddPCR™ Mutation Assay: BRAF p.V600R c.1798_1799GT>AG, Human, Homo sapiens	https://www.bio-rad.com/digital-assays/assay-detail/dHsaMDV2010037, accessed on 4 December 2023
ddPCR EGFR Exon 19 Deletions Screening Kit #12002392	ddPCR™ Mutation Assay: EGFR p.E746_T751>I c.2235_2252>AAT, Human, Homo sapiens	https://www.bio-rad.com/digital- assays/assay-detail/dHsaMDS391737271, accessed on 4 December 2023
	ddPCR™ Mutation Assay: EGFR p.E746_A750delELREA c.2235_2249del15, Human, Homo sapiens	https://www.bio-rad.com/digital-assays/assay-detail/dHsaMDV2010039, accessed on 4 December 2023
	ddPCR™ Mutation Assay: EGFR p.E746_A750delELREA c.2236_2250del15, Human, Homo sapiens	https://www.bio-rad.com/digital- assays/assay-detail/dHsaMDS542127747, accessed on 4 December 2023
	ddPCR™ Mutation Assay: EGFR p.L747_T751>Q c.2238_2252>GCA, Human, Homo sapiens	https://www.bio-rad.com/digital- assays/assay-detail/dHsaMDS435546404, accessed on 4 December 2023
	ddPCR™ Mutation Assay: EGFR p.E746_S752>D, Human, Homo sapiens	https://www.bio-rad.com/digital- assays/assay-detail/dHsaMDS529140399, accessed on 4 December 2023
	ddPCR™ Mutation Assay: EGFR p.L747_T751>Q c.2239_2253>CAA, Human, Homo sapiens	https://www.bio-rad.com/digital- assays/assay-detail/dHsaMDS703176693, accessed on 4 December 2023
	ddPCR™ Mutation Assay: EGFR p.L747_T751>P c.2239_2251>C, Human, Homo sapiens	https://www.bio-rad.com/digital-assays/assay-detail/dHsaMDV2516890, accessed on 4 December 2023
	ddPCR™ Mutation Assay: EGFR p.L747_P753>Q, Human, Homo sapiens	https://www.bio-rad.com/digital- assays/assay-detail/dHsaMDS196056967, accessed on 4 December 2023
	ddPCR™ Mutation Assay: EGFR p.L747_T751>Q c.2239_2252>CA, Human, Homo sapiens	https://www.bio-rad.com/digital- assays/assay-detail/dHsaMDS651224081, accessed on 4 December 2023
	ddPCR™ Mutation Assay: EGFR p.L747_S752delLREATS, Human, Homo sapiens	https://www.bio-rad.com/digital-assays/assay-detail/dHsaMDV2516758, accessed on 4 December 2023
	ddPCR™ Mutation Assay: EGFR p.L747_A750>P c.2239_2248TTAAGAGAAG>C, Human, Homo sapiens	https://www.bio-rad.com/digital-assays/assay-detail/dHsaMDV2516748, accessed on 4 December 2023
	ddPCR™ Mutation Assay: EGFR p.L747_T751delLREAT c.2239_2253del15, Human, Homo sapiens	https://www.bio-rad.com/digital-assays/assay-detail/dHsaMDV2516752, accessed on 4 December 2023
	ddPCR™ Mutation Assay: EGFR p.L747_E749delLRE, Human, Homo sapiens	https://www.bio-rad.com/digital- assays/assay-detail/dHsaMDS88236242, accessed on 4 December 2023
	ddPCR™ Mutation Assay: EGFR p.L747_T751delLREAT c.2240_2254del15, Human, Homo sapiens	https://www.bio-rad.com/digital- assays/assay-detail/dHsaMDS778667043, accessed on 4 December 2023
	ddPCR™ Mutation Assay: EGFR p.L747_P753>S c.2240_2257del18, Human, Homo sapiens	https://www.bio-rad.com/digital-assays/assay-detail/dHsaMDV2510546, accessed on 4 December 2023

ddPCR™ NRAS Q61 Screening Kit #12001006	ddPCR™ Mutation Assay: NRAS p.Q61K c.181C>A, Human, Homo sapiens	https://www.bio-rad.com/digital- assays/assay-detail/dHsaMDV2010067, accessed on 4 December 2023
	ddPCR™ Mutation Assay: NRAS p.Q61L c.182A>T, Human, Homo sapiens	https://www.bio-rad.com/digital- assays/assay-detail/dHsaMDV2010069, accessed on 4 December 2023
	ddPCR™ Mutation Assay: NRAS p.Q61R c.182A>G, Human, Homo sapiens	https://www.bio-rad.com/digital- assays/assay-detail/dHsaMDV2010071, accessed on 4 December 2023
	ddPCR™ Mutation Assay: NRAS p.Q61H c.183A>T, Human, Homo sapiens	https://www.bio-rad.com/digital- assays/assay-detail/dHsaMDV2010065, accessed on 4 December 2023
	ddPCR™ Mutation Assay: NRAS p.Q61H c.183A>C, Human, Homo sapiens	https://www.bio-rad.com/digital- assays/assay-detail/dHsaMDV2510578, accessed on 4 December 2023
ddPCR™ NRAS G12/G13 Screening Kit #12001627	ddPCR™ Mutation Assay: NRAS p.G12A, Human, Homo sapiens	https://www.bio-rad.com/digital- assays/assay-detail/dHsaMDS42165742, accessed on 4 December 2023
	ddPCR™ Mutation Assay: NRAS p.G12C, Human, Homo sapiens	https://www.bio-rad.com/digital- assays/assay-detail/dHsaMDV2510530, accessed on 4 December 2023
	ddPCR™ Mutation Assay: NRAS p.G12D, Human, Homo sapiens	https://www.bio-rad.com/digital- assays/assay-detail/dHsaMDV2010095, accessed on 4 December 2023
	ddPCR™ Mutation Assay: NRAS p.G12S, Human, Homo sapiens	https://www.bio-rad.com/digital- assays/assay-detail/dHsaMDV2010093, accessed on 4 December 2023
	ddPCR™ Mutation Assay: NRAS p.G12V, Human, Homo sapiens	https://www.bio-rad.com/digital- assays/assay-detail/dHsaMDV2510528, accessed on 4 December 2023
	ddPCR™ Mutation Assay: NRAS p.G13D, Human, Homo sapiens	https://www.bio-rad.com/digital- assays/assay-detail/dHsaMDV2510526, accessed on 4 December 2023
	ddPCR™ Mutation Assay: NRAS p.G13R, Human, Homo sapiens	https://www.bio-rad.com/digital- assays/assay-detail/dHsaMDV2510534, accessed on 4 December 2023
	ddPCR™ Mutation Assay: NRAS p.G13V c.38G>T, Human, Homo sapiens	https://www.bio-rad.com/digital- assays/assay-detail/dHsaMDV2510524, accessed on 4 December 2023
ddPCR™ KRAS Q61 Screening Kit #12001626	ddPCR™ Mutation Assay: KRAS p.Q61K c.181C>A, Human, Homo sapiens	https://www.bio-rad.com/digital- assays/assay-detail/dHsaIS2501862, accessed on 4 December 2023
	ddPCR™ Mutation Assay: KRAS p.Q61L c.182A>T, Human, Homo sapiens	https://www.bio-rad.com/digital- assays/assay-detail/dHsaMDV2010101, accessed on 4 December 2023
	ddPCR™ Mutation Assay: KRAS p.Q61R c.182A>G, Human, Homo sapiens	https://www.bio-rad.com/digital- assays/assay-detail/dHsaMDV2010135, accessed on 4 December 2023
	ddPCR™ Mutation Assay: KRAS p.Q61H c.183A>T, Human, Homo sapiens	https://www.bio-rad.com/digital- assays/assay-detail/dHsaMDV2010131, accessed on 4 December 2023
	ddPCR™ Mutation Assay: KRAS p.Q61H c.183A>C, Human, Homo sapiens	https://www.bio-rad.com/digital- assays/assay-detail/dHsaMDV2010133, accessed on 4 December 2023

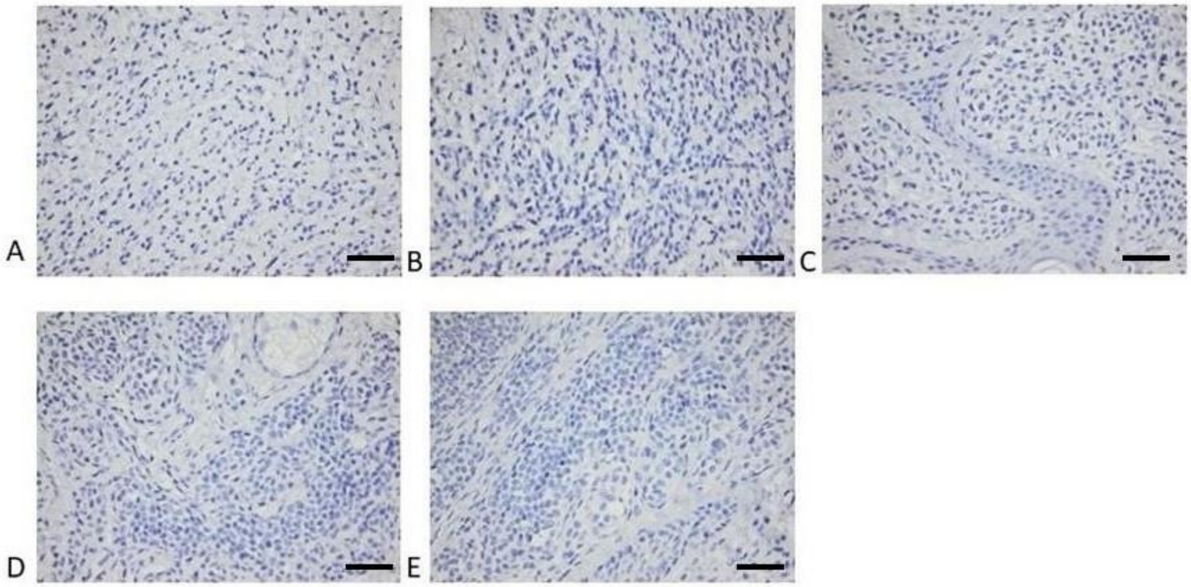

Figure S2. Negative controls for IHC of the following protein expressions: p16, p21,p53, bcl2, cyclin D1. A. Negative control for p16 x 400. B. Negative control for p21 x 400. C Negative control for bcl2 x 400. D. Negative control for p53 x 400. E. Negative control for cyclin D1 x 400 (scale bar = 50 μm).

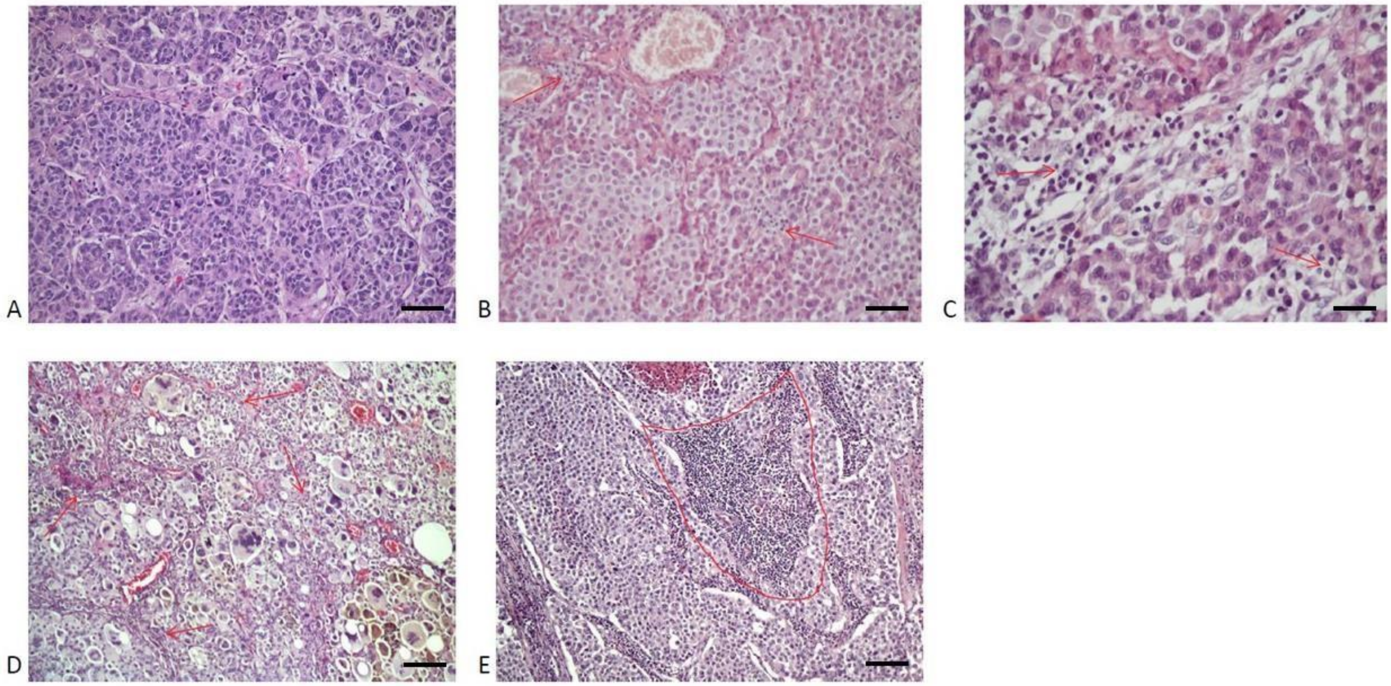

Figure S3. (A) Nodular melanoma. No intratumor lymphocytic infiltrate. HE x 200 (scale bar = 100 μm). (B) Nodular melanoma. Mild lymphocytic infiltrate (TIL non-brisk). HE x 200 (scale bar = 100 μm). (C) Nodular melanoma. Mild lymphocytic infiltrate (TIL non- brisk). HE x 400 (scale bar = 50 μm). (D) Nodular melanoma. Abundant lymphocytic infiltrate (TIL brisk). HE x 200 (scale bar = 100 μm). (E) Nodular melanoma. Abundant lymphocytic infiltrate (TIL brisk). HE x 100 (scale bar = 200 μm). Red arrows depict infiltrated tumor areas. The red free form in Figure S3-E also depicts a large area of TILs.

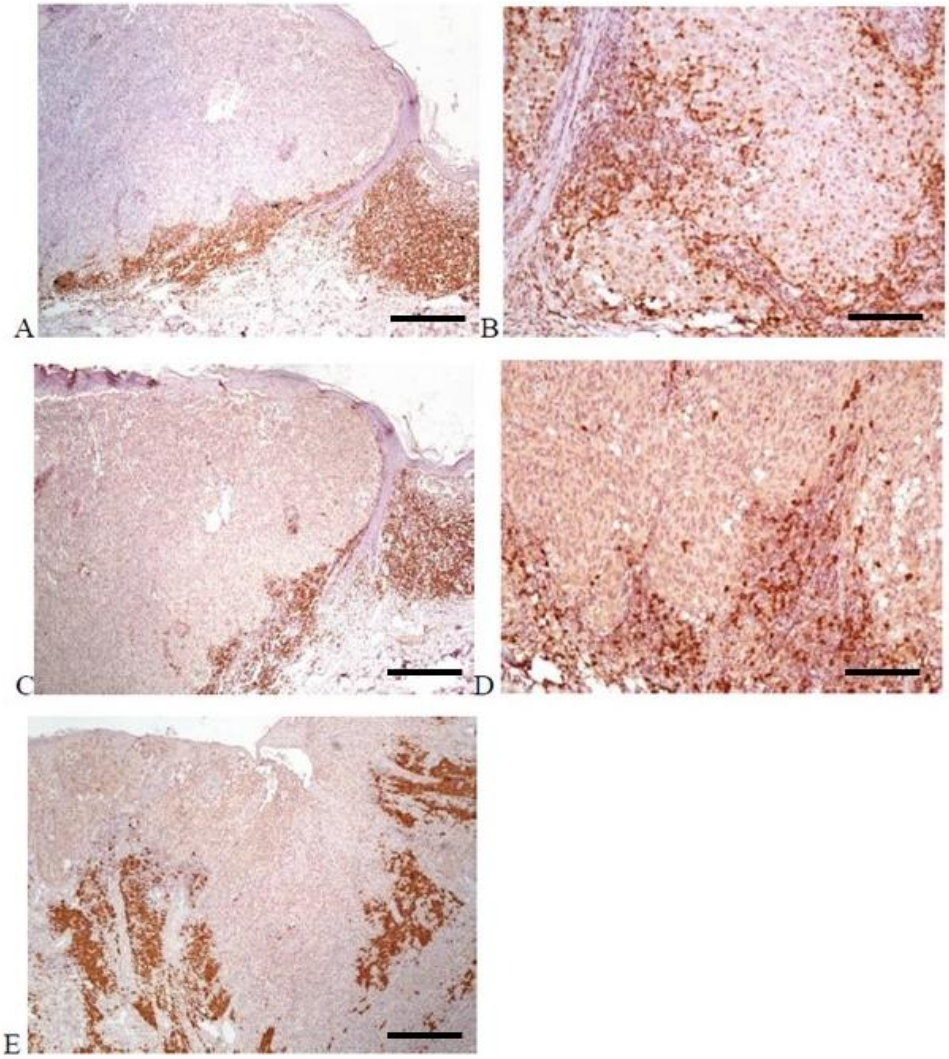

Figure S4. Assessment of the inflammatory infiltrate of cutaneous tumors. (A) Superficial spreading melanoma with numerous CD3+ T cells (non-brisk TIL infiltrate), CD3 x 40 (scale bar = 500 μm). (B) Superficial spreading melanoma with increased CD4+ T cell infiltration within tumor mass and in the peritumoral stroma (brisk TIL infiltrate), CD4 x 100 (scale bar = 200 μm). (C) Superficial spreading melanoma with numerous CD20+ B lymphocytes (non-brisk TIL infiltrate), CD20 x 40 (scale bar = 500 μm). (D) Superficial spreading melanoma with numerous B cells infiltrating the whole base of the invasive tumor (brisk TIL infiltrate), CD20 x 100 (scale bar = 200 μm). (E) Superficial spreading melanoma with multiple CD8+ T cell lymphocytes infiltrating the entire base of the invasive tumor (brisk TIL infiltrate), CD8 x 40 (scale bar = 500 μm). The brown chromogen deposits indicate positive staining. All the presented tumors have high peritumoral inflammation, but the intratumoral status is non-brisk or brisk.
